# Toward an Improvement of the Analysis of Neural Coding

**DOI:** 10.3389/fninf.2017.00077

**Published:** 2018-01-10

**Authors:** Javier Alegre-Cortés, Cristina Soto-Sánchez, Ana L. Albarracín, Fernando D. Farfán, Mikel Val-Calvo, José M. Ferrandez, Eduardo Fernandez

**Affiliations:** ^1^Neuroprosthetics and Visual Rehabilitation Research Unit, Bioengineering Institute, Miguel Hernández University, Alicante, Spain; ^2^Biomedical Research Networking Center in Bioengineering, Biomaterials and Nanomedicine, Madrid, Spain; ^3^Biotechnology Department, University of Alicante, Alicante, Spain; ^4^Laboratorio de Medios e Interfases, Departamento de Bioingeniería, Facultad de Ciencias Exactas y Tecnología, Universidad Nacional de Tucumán, Tucumán, Argentina; ^5^Departamento de Bioingeniería, Instituto Superior de Investigaciones Biológicas, Consejo Nacional de Investigaciones Científicas y Técnicas, Tucumán, Argentina; ^6^Departamento de Electrónica, Tecnología de Computadoras, Universidad Politécnica de Cartagena, Cartagena, Spain

**Keywords:** neuronal coding, non-linear signals, NA-MEMD, machine learning classification, single trial classification

## Abstract

Machine learning and artificial intelligence have strong roots on principles of neural computation. Some examples are the structure of the first perceptron, inspired in the retina, neuroprosthetics based on ganglion cell recordings or Hopfield networks. In addition, machine learning provides a powerful set of tools to analyze neural data, which has already proved its efficacy in so distant fields of research as speech recognition, behavioral states classification, or LFP recordings. However, despite the huge technological advances in neural data reduction of dimensionality, pattern selection, and clustering during the last years, there has not been a proportional development of the analytical tools used for Time–Frequency (T–F) analysis in neuroscience. Bearing this in mind, we introduce the convenience of using non-linear, non-stationary tools, EMD algorithms in particular, for the transformation of the oscillatory neural data (EEG, EMG, spike oscillations…) into the T–F domain prior to its analysis with machine learning tools. We support that to achieve meaningful conclusions, the transformed data we analyze has to be as faithful as possible to the original recording, so that the transformations forced into the data due to restrictions in the T–F computation are not extended to the results of the machine learning analysis. Moreover, bioinspired computation such as brain–machine interface may be enriched from a more precise definition of neuronal coding where non-linearities of the neuronal dynamics are considered.

## Introduction

The mutual benefits of the interplay between natural and artificial computation are well-known. Moreover, the increasing volume and complexity of the generated data in neuroscience exceeds the capacity of classical analysis, and they are becoming more and more difficult to analyze. In this scenario, the emergence of artificial computation and machine learning (ML) techniques is becoming crucial for the interpretation and analysis of these complex data. Some examples are the interaction between networks and behavior (Bathellier et al., 2012), stimulus coding (Nikolić et al., 2009; Klampfl et al., 2012), population dynamics in neural networks (Buonomano and Merzenich, 1995), classification of behavioral states (Kabra et al., 2012), and spike sorting procedures (Bongard et al., 2014; Carlson et al., 2014; Dimitriadis et al., 2016). On the other hand artificial computation has received inspiration from neuroscience since the first artificial neuron developed in the 40's (McCulloch and Pitts, 1943), continuing to the first perceptron, inspired in the circuitry of the retina (Rosenblatt, 1957), Hopfield networks (Hopfield, 1982), or Self-Organizing maps (Kohonen, 1982) and is still widely present nowadays.

The scope of this perspectives paper is to highlight the reliability and usefulness of ML techniques for the analysis of electrophysiological recordings. In particular, we will address the manipulation of the data prior to its analysis and classification, specifically regarding to Time–Frequency (T–F) features. In this framework we think that T–F analysis tools have not been as extensively implemented as other ML algorithms in neuroscience research. To facilitate the analysis of relevant T–F information using ML analysis, we propose to use Empirical Mode Decomposition data-driven algorithms (Huang et al., 1998, EMD) to extract the relevant T–F features to be studied. This procedure is widely used in signal analysis and has been proved successfully in the analysis of electrophysiological data (Li, 2006; Huang et al., 2013; Hu and Liang, 2014; Al-Subari et al., 2015; Alegre-Cortés et al., 2016); nevertheless, they have not yet become of common use and are sparsely found in neuroscience publications. As a result, we still use linear and stationary techniques that are unavoidably biasing and blurring relevant information, since they are not able to accurately depict the intermittency and non-linearity of the data. This approach usually leads to the underperformance of classification or pattern extraction using ML algorithms, hence limits the strength of the posterior analysis (Mandic et al., 2013). The general idea behind this suggestion is that a more precise transformation into the T–F domains of the data will improve the result of the classification and/or search for patterns in the data performed by the ML algorithms.

## Synergy between EMDs, machine learning, and brain processes

Most of the brain processes are non-linear and non-stationary. Hence, the selected analytical tools require the capability to deal with these properties of the data. EMDs (Huang et al., 1998) are data driven algorithms designed to extract oscillatory information without its projection onto any predefined function, converting the original signal into a sum of oscillatory components called Intrinsic Mode Functions (IMFs). In this way they facilitate the extraction of meaningful information from the data without temporal or waveform restrictions, usually using the Hilbert transform (Huang et al., 1998). In addition, there exist multivariate variations of these algorithms (Rehman and Mandic, 2010; Ur Rehman and Mandic, 2011) that allow a simultaneous decomposition of multiple recorded neuronal signals. This is possible thanks to the simultaneous decomposition of all dimensions of the data, which ensures the same number of IMFs containing the information in the same frequency ranges (Rehman and Mandic, 2010). Thus, thanks to the advantages of EMD algorithms over classic linear analysis, they are being increasingly used in neuronal analysis (Liang et al., 2005; Huang et al., 2013; Al-Subari et al., 2015; Alegre-Cortés et al., 2016), and they are helping us to achieve a better understanding of the oscillatory properties of neuronal activity (Buzsáki and Draguhn, 2004).

Despite the advantages of this approach, we should take into account that EMD algorithms increase the dimensionality of the data, since they convert the original signal in a set of IMFs. Hence these procedures increase the difficulties in the management of the data to extract useful results or perform any desired classification.

In this context, ML techniques are the perfect tools to analyze and classify the decomposed neuronal activity. ML is a subfield of statistics and computer science, which takes advantage of the power of computers to perform iterative computations to identify the existing patterns on the data to make future models and predictions. Furthermore, the projection of the data into a higher dimensional space provides an additional advantage, since it helps to improve discrimination (Cover, 1965).

To support these ideas and the advantages of the proposed approach, we will briefly introduce a couple of real examples based on different experimental approaches and electrophysiological techniques.

## Texture discrimination from vibrissal nerve recordings

**Figure 1:**
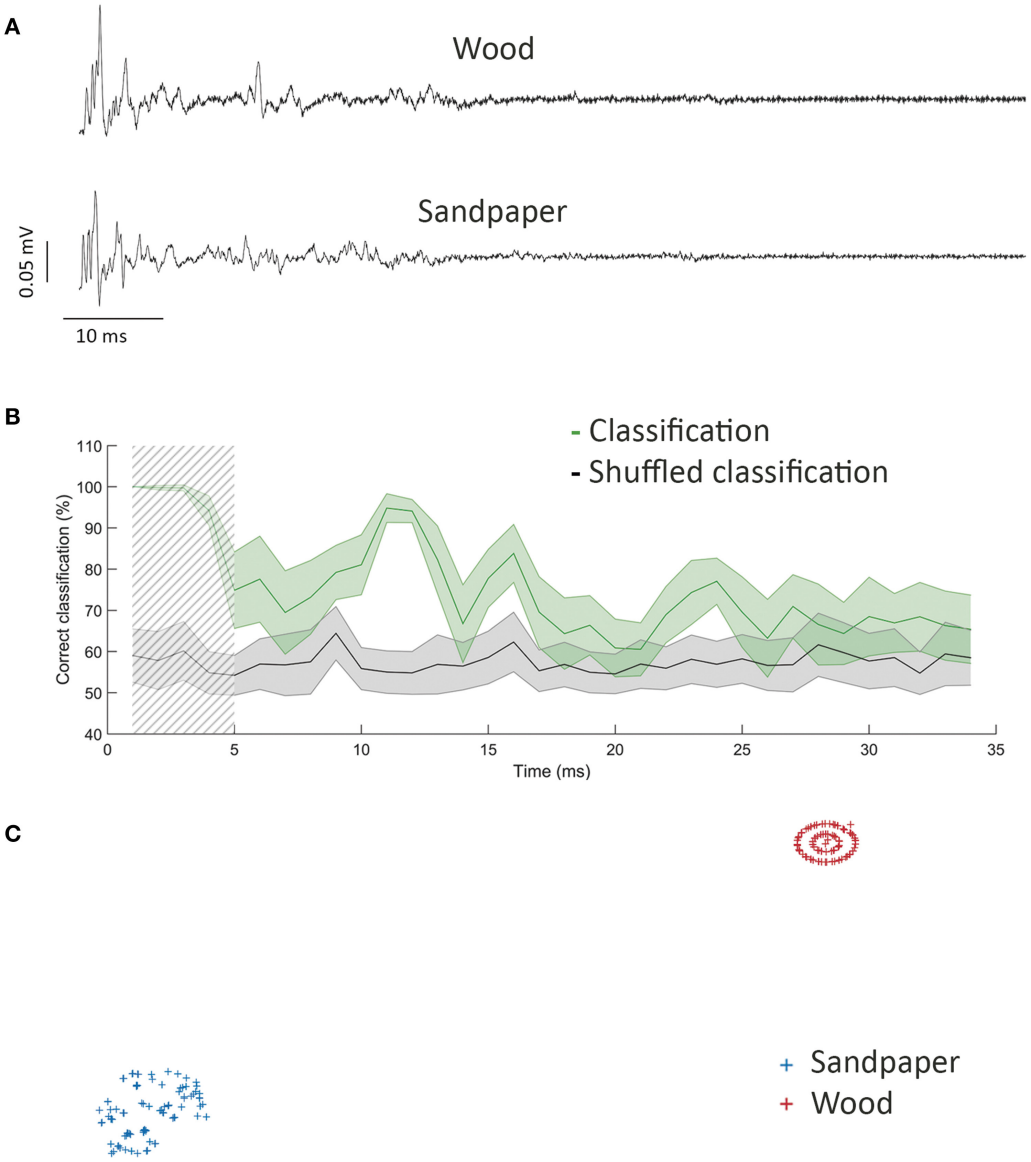
Texture discrimination using NA-MEMD plus MLP. **(A)** Mean vibrissal nerve response to sweeping wood (top) and sandpaper (bottom). **(B)** Percentage of correct classification (green) and classification after shuffling (gray). Shadow square represents maximum discrimination window, used in **(C)**. Error displayed as s.e.m. **(C)** t-SNE representation of vibrissal nerve activity during the first 5 ms of the response.

The first example are electrophysiological recordings from rat vibrissal nerve during a texture discrimination task (see Albarracín et al., 2006 for details). Previous work with this data (Lucianna et al., 2016) using linear techniques for T–F features extraction (Root Mean Square value to estimate signal energy and Burg parametric estimation method to compute the Power Spectrum Density) and a simple perceptron (Hertz et al., 1991) concluded that five sweeps were required for an adequate texture classification. To probe our thoughts, we performed a similar analysis on the discrimination of the pair of materials of hardest discrimination, wood vs. L1000 sandpaper (Figure 1A), using information from single sweeps on the surface. Previous results on these pair of textures had described that a single swept provided just 70% correct texture classification and had great variability.

We decomposed the data using Noise Assisted Multivariate Empirical Mode Decomposition (NA-MEMD, Ur Rehman and Mandic, 2011) to obtain the T–F spectrum of the response to each texture (see Alegre-Cortés et al., 2016 for details). Standard stopping criterion is described in Rilling et al. (2003). The obtained mean amplitude and mean IF of the different IMFs between 115 and 384 Hz were used to train a multilayer perceptron (MLP) to perform the classification (Cybenko, 1989). We used a single hidden layer of 14 neurons and scaled conjugated gradient as supervised training algorithm (Powell, 1977). We repeated this analysis on a sliding window to compute the temporal profile of discrimination between these textures during 100 ms after stimulus offset. To prevent from biased results due to the finite number of experiments, we shuffled texture across our data to determine the average error in classification. This process was repeated 100 times in each window.

Figure 1 shows the main results. Discrimination was maximal (99.5 ± 0.5%, shadow square, Figure 1B) during a 5 ms window starting 5 ms after stimulus offset. Discrimination performance decreased during time, but a second peak of discrimination was seen 15–20 ms after stimulation, coinciding in time with the second contact with the surface during the withdrawal of the whisker. When we compared this maximum value of discrimination (Figure 1B) with the previous results obtained on the discrimination on these dataset (≈70% on average, Lucianna et al., 2016) we confirmed an evident increase in texture discrimination thanks to the combined use of NA-MEMD followed by ML classification. Moreover, the classification was based on single-trial recordings and was shown to had almost no variability in the peak of discrimination (Figure 1B), providing an additional improvement over previous results.

We used the t-distributed stochastic neighbor embedding algorithm (t-SNE) (van der Maaten and Hinton, 2008) as an additional ML technique to differentiate the vibrissal nerve response to the different stimulating textures (wood and sandpaper), starting from the same parameters we used to train the MLP in a time window of 5 ms length starting 5 ms after stimulation, coinciding with maximum discrimination in Figure 1B. This technique is useful to reduce the dimensionality of the data and allowed us to classify our complex data into two different and well-separated clusters, each one corresponding to one of the stimulating textures: wood and sandpaper (Figure 1C).

## Stimulation electrode discrimination from multielectrode primary cortical neurons culture recordings

**Figure 2:**
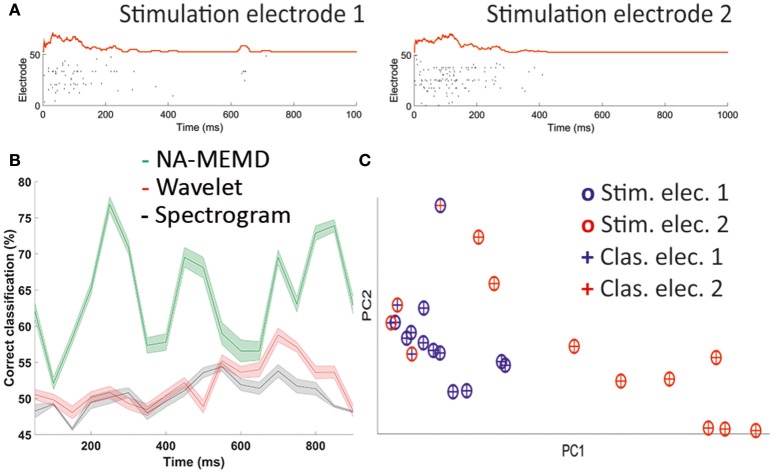
Stimulation electrode discrimination. **(A)** Example of a single stimulation in each stimulation electrode. Raster plot of the whole electrode and mean activity vector. **(B)** Percentage of correct classification using NA-MEMD (green), Morlet wavelet (red), and spectrogram (gray). Error displayed as s.e.m. **(C)** Distribution of individual trials after using PCA (crosses) and clusterization using DBSCAN algorithm (circles).

To further illustrate the power and potential of this approach, we carried out an additional analysis of simultaneous recordings in primary cortical neurons cultures (see Calvo et al., 2016 for details). Briefly, embryonic primary cortical neurons were cultured on a multielectrode array; then, population activity was recorded simultaneously at 60 points of the culture while electrically stimulated in two different electrodes of the array (Figure 2A). We decomposed the averaged activity present in the electrode to obtain the mean oscillatory activity during 100 stimulations in each of the stimulation electrodes independently using NA-MEMD. Then, we extracted different values of mean amplitude and mean IF at different T–F windows (IMFs ranging from 30 to 90 Hz) to train a MLP to discriminate the stimulation electrode, from the recorded activity when a minimum number of spikes were evoked in the whole response window. We used a single hidden layer of 15 neurons and scaled conjugated gradient as supervised training algorithm. An equivalent shuffling procedure was done to subtract chance-level classification. This process was repeated 100 times.

Once we subtracted chance-level classification, stimulation electrode had its maximum discrimination peak 200–300 ms after stimulation, exceeding 75% successful classification when we used NA-MEMD as the feature extraction tool (Figure 2B). Therefore, we were able to discriminate the electrode on which the unique stimulation had occurred analyzing the oscillatory properties of the generated response. This was not possible when we extracted the T–F features to train the MLP using either spectrogram or wavelet (Morlet) analysis. When we used these linear techniques, stimulation electrode classification was similar to chance-level classification (Figure 2B).

We performed an additional analysis applying a density-based algorithm for discovering clusters in large spatial databases with noise named DBSCAN that is designed to discover clusters of arbitrary shape (Ester et al., 1996). This algorithm was applied to the extracted parameters during the window of maximum discrimination using NA-MEMD in Figure 2B (200–300 ms after stimulus onset). We found two clusters (Figure 2C), corresponding to the two stimulation electrodes. A total of 83% of the trials were in the correct clusters, in clear coincidence with the mean percentage of correct classification of the MLP in that window of time before the subtraction of the chance-level classification.

## Concluding remarks

Over the last decade, many technical and conceptual issues related with the analysis of neuronal recordings have been addressed, but there are still some problems related with the analysis of T–F data. We suggest that a combination of T–F signal decomposition via EMD algorithms (NA-MEMD, in our case) plus a posterior classification of the obtained parameters using ML techniques are powerful tools in this framework. Therefore, the implementation of this combination of analytical tools in the daily neuroscience research would improve the information extracted from the recorded single or multiple neuronal activities and, in ultimate extent, increase our understanding of the nervous system. Furthermore, although more studies are still needed, these tools could be also useful for a better understanding of some pathological processes of the brain.

## Ethics statement

All the procedures carried out at the Institute for Biological Research (INSIBIO)/Instituto Superior de Investigaciones Biológicas, were in accordance with the recommendations of the Guide for the Care and Use of Laboratory Animals (National Research Council, NRC). All the experimental procedures carried out at the Miguel Hernandez University were conformed to the directive 2010/63/EU of the European Parliament and of the Council, and the RD 53/2013 Spanish regulation on the protection of animals use for scientific purposes and approved by the Miguel Hernandez University Committee for Animal use in Laboratory.

## Author contributions

Data have been provided by AA, FF, and MV-C. Data have been analyzed by JA-C. Paper was written by JA-C, CS-S, JF, and EF.

### Conflict of interest statement

The authors declare that the research was conducted in the absence of any commercial or financial relationships that could be construed as a potential conflict of interest.
